# List of reviewers

**DOI:** 10.1192/bji.2022.17

**Published:** 2022-08

We would like to thank those who contributed as peer reviewers in the period from January 2021–December 2021. Their anonymous work for the journal forms the foundation to its success and is highly appreciated.

Ahmed Abdelgawad

Mohammed Abousaleh

Adeniyi Adetoki

Keyrellous Adib

Frances Adiukwu

Raja Adnan Ahmed

Anthony Akenzua

Hasanen Al-Taiar

Mohammed Al-Uzri

Elham Aldouri

Numan Ali

Nadim Almoshmosh

Humma Andleeb

Chittaranjan Andrade

Bintang Arroyantri Prananjaya

Oscar Arteaga

Suraj Bhushal

Brad Booth

Nick Bouras

John Bradford

David Bukusi

Alfonso Cesar

Rakesh Chadda

Santosh Chaturvedi

Poom Chompoosri

Nikos Christodoulou

John Cookson

Emily Corner

John Cox

Subodh Dave

June De Leon

Raman Deep

Saleh Dhumad

Richard Duffy

Allen Dyer

Johann Ebenezer

Joseph El-Khoury

Emily Finch

Jane Fisher

Laura Freeman

Alejandra Fuentes

Vivek Furtado

Gaurish Gaunekar

Suhaila Ghuloum

Pabasari Ginige

Sundar Gnanavel

John Gunn

Dr Susham Gupta

Margaret Hogan

Guesche Huebner

Peter Hughes

Matías Irarrazaval

Rachel Jenkins

Stefan Jerotic

Ruta Karaliuniene

Elie Karam

Kenan Karbeyaz

Shireen Kassam

Preeti Khanna

Yasser Khazaal

Petros Lekkos

Jeffrey Looi

Jan Looman

Sharika Mahato

Sood Mamta

Mohammed A Mamun

Sofia Manolesou

Dmytro Martsenkovsky

Rosalind McAlpine

Alexandra Moore

David Ndetei

Naresh Nebhinani

Tarek Okasha

Ramanand Pandit

Ravi Paul

Victor Pereira-Sanchez

Dr Sayuri Perera

Mariana Pinto da Costa

Samir Prahraj

Victor Puac-Polanco

Yugesh Rai

Harshini Rajapakse

Rodrigo Ramalho

David Reiss

Hashim Reza

Maria Rogdaki

Julia Ronder

Rukhsana Roshan

Stella Rosson

Khalid Saeed

John Saunders

Aspasia Serdari

Terence Sharkey

Thanapal Sivakumar

Pau Soldevila-Matías

Ramya Srinivasan

Thomas Steare

Tharwat Sulaiman

Johanne Sundby

Marcus Tan

Allan Tasman

Jeevan Thapa

Tarje Tinderholt

Derek Tracy

Christos Tsopelas

Rohit Verma

Elsawy Wafaa

Nicola Warren

Chamara Wijesinghe

James Woollard

Huma Zill-e

